# Experimental comparison of relative RT-qPCR quantification approaches for gene expression studies in poplar

**DOI:** 10.1186/1471-2199-11-57

**Published:** 2010-08-11

**Authors:** Nicole Regier, Beat Frey

**Affiliations:** 1 Swiss Federal Research Institute WSL, Zürcherstrasse 111, 8903 Birmensdorf, Switzerland; 2 Current Address: Université de Genève, Institut Forel, CP 416, 1290 Versoix, Switzerland

## Abstract

**Background:**

RT-qPCR is a powerful tool for analysing gene expression. It depends on measuring the increase in fluorescence emitted by a DNA-specific dye during the PCR reaction. For relative quantification, where the expression of a target gene is measured in relation to one or multiple reference genes, various mathematical approaches are published. The results of relative quantification can be considerably influenced by the chosen method.

**Results:**

We quantified gene expression of superoxide dismutase (*SOD*) and ascorbate peroxidase (*APX*) in the roots of two black poplar clones, 58-861 and Poli, which were subjected to drought stress. After proving the chosen reference genes actin (*ACT*), elongation factor 1 (*EF1*) and ubiquitin (*UBQ*) to be constantly expressed in the different watering regimes, we applied different approaches for relative quantification to the same raw fluorescence data. The results obtained using the comparative Cq method, LinRegPCR, qBase software and the Pfaffl model showed a good correlation, whereas calculation according to the Liu and Saint method produced highly variable results. However, it has been shown that the most reliable approach for calculation of the amplification efficiency is using the mean increase in fluorescence during PCR in each individual reaction. Accordingly, we could improve the quality of our results by applying the mean amplification efficiencies for each amplicon to the Liu and Saint method.

**Conclusions:**

As we could show that gene expression results can vary depending on the approach used for quantification, we recommend to carefully evaluate different quantification approaches before using them in studies analysing gene expression.

## Background

RT-qPCR is a widely used method for analysing gene expression. It has been developed by combining PCR with fluorescent techniques [1,2]. It depends on collecting data throughout the PCR amplification, which is achieved by monitoring the increase in fluorescence intensity of a specific fluorescence dye, which correlates to the increase in PCR product concentration. The major progress of qPCR is that quantification does not have to be done in the plateau phase of amplification, which is a disadvantage of previous quantification methods [1].

PCR can be divided into four major phases: linear ground phase, early exponential phase, log-linear phase and plateau phase [3]. During the linear ground phase, only background fluorescence is detected. The early exponential phase starts when the amount of fluorescence is significantly higher than the background. During the log-linear phase, when PCR has reached its optimal amplification period, the amount of fluorescence rises exponentially. In an ideal reaction the PCR products double after every cycle. Finally, when the reaction components become limited, the plateau phase is reached and the fluorescence does not increase anymore [4].

Relative quantification of RT-qPCR is used to detect changes in expression of the genes of interest relative to a reference gene, which is usually a housekeeping gene. Early RT-qPCR studies have assumed that housekeeping genes are expressed constantly over a wide range of conditions. Later studies have shown that their expression stability should be proven before choosing them as references, and proposed to use not only one, but several reference genes as internal controls [5,6].

Many approaches are available for relative quantification of gene expression. Most of them depend on the principle to define a threshold at which the PCR product fluorescence rises over the background fluorescence. The number of cycles needed until this threshold is reached, depending on the amount of template in a sample, is usually called Cq - the higher the template amount, the lower is the Cq value. Widely used approaches depending on this principle are the 2^-ΔΔCq ^or comparative Cq method [7], the Pfaffl model [8], or qBase software [9]. While the comparative Cq method assumes the same amplification efficiency for all amplicons, other methods use serial dilutions of the samples to determine the amplification efficiencies from the increase in the Cq value with decreasing cDNA input [10]. Another approach is to determine the amplification efficiency or starting template amount from the increase in fluorescence during the PCR reaction [11,12].

The aim of this study was to evaluate, whether the different approaches for relative quantification of RT-qPCR generate comparable results. We wanted to find suitable reference genes for normalization of gene expression in a study investigating drought responses of poplar [13,14] and to test whether the tested methods differ in reliability and suitability for our approach of quantifying gene expression. For this test, ascorbate peroxidase (*APX*) and superoxide dismutase (*SOD*) were used as target genes, as they play an important role in oxidative stress defence in plants subjected to drought. Actin (*ACT*), elongation factor 1 (*EF1*) and ubiquitin (*UBQ*) were used as reference genes.

## Results and Discussion

### Evaluation of expression stability of the reference genes

**Figure 1:**
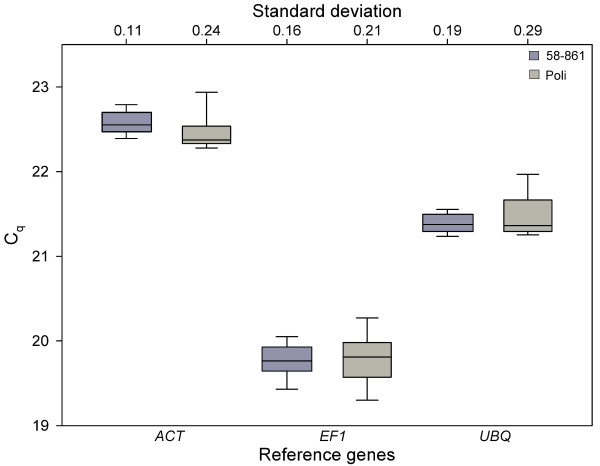
**Expression stability of the reference genes according to Reid et al (2006) **[6]. Cq values of the reference genes *ACT*, *EF1 *and *UBQ *are shown over all treatments separately for each clone. Boxes represent 25 and 75 quartiles, Whisker caps indicate 10 and 90 percentiles, and medians are shown by the line. Standard deviations of the Cq values are given separately for each clone. *ACT*, actin; *EF1*, elongation factor 1; *UBQ*, ubiquitin; n = 9.

As the reference genes are used to normalize expression of the target genes, they must be unaffected among the samples to be compared. We tested the expression stability of the reference genes by three different approaches presented in the literature [5,6,15]. The first depends on comparing the Cq values of all samples used in an experiment and to calculate the standard deviation [6]. The variation in reference gene expression between the different treatments and clones was very low (Figure 1), and we did not find any significant differences between the clones. Therefore all three reference genes were assumed to be suitable for normalising gene expression in our experiment.

**Figure 2:**
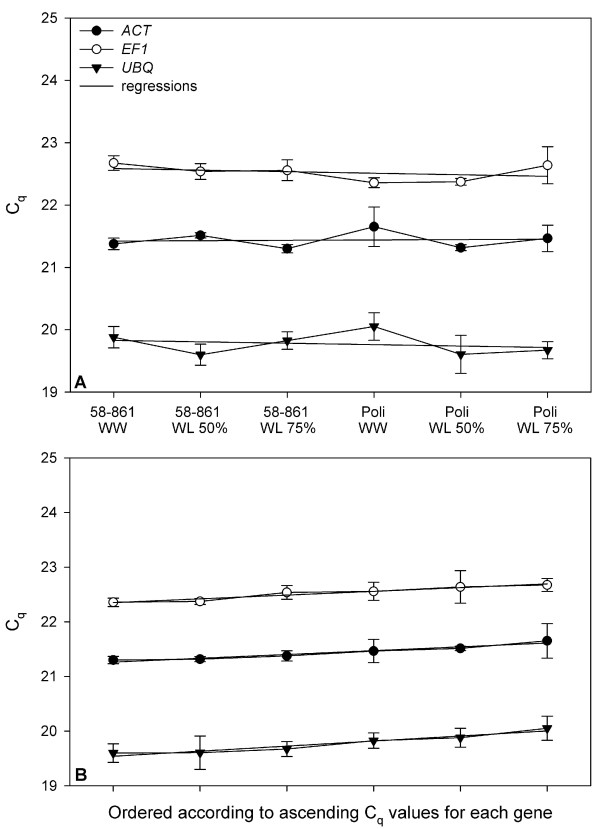
**Expression stability of the reference genes according to Brunner et al (2004) **[5]. Regression of Cq values against sample was calculated. (A) Samples were ordered according to clone and treatment, (B) samples were ordered according to ascending Cq values. For genes with high expression stability, the slope of the regression line is low and the fit is close. WW, well-watered; WL 50%, water limited (50% less water than control); WL 75%, water limited (75% less water than control). *ACT*, actin; *EF1*, elongation factor 1; *UBQ*, ubiquitin; n = 3.

The second approach assessed expression stability by the slope of the regression line when the Cq values are plotted against the respective samples. The lower the slope is, the more stably the gene is expressed [5]. As the authors do not give any advice on how to order the samples for regression, we tested two approaches: first we ordered the samples randomly according to poplar clone and treatment, second we ordered them according to the Cq values in order to obtain the maximal possible slopes of the regression lines (Figure 2). All reference genes showed high expression stability, the slopes of all regression lines were close to zero. Namely, they were 0.006 for *ACT*, and -0.02 for *EF1 *and *UBQ*, respectively, when ordering the samples randomly. When ordering the samples according to ascending Cq values, which maximizes the slopes of the regression lines, the fit was closer than when ordering the samples randomly (R^2 ^= 0.94 for all genes), but the slopes were still low (0.07 for *ACT *and *EF1*, respectively, and 0.09 for *UBQ*), indicating that expression of the genes was not altered by the different drought treatments. Furthermore, we did not find any significant differences between the Cq values of the different clone/treatment combinations.

As third approach for calculation of reference gene expression stability we used the NormFinder software [15]. It revealed expression stabilities of 0.105 for *ACT*, 0.142 for *EF1 *and 0.099 for *UBQ*. Comparison with other studies using this program [16-18] revealed that our reference genes had comparable or even higher expression stability than the ones reported in the literature. This high stability revealed with all applied evaluation approaches [5,6,15] might be due to the low number of sample types (expression analysis only in roots, two clones and three treatments, which gives a final number of 6 sample types) as compared to the other studies.

Interestingly, the three approaches did not identify the same reference gene to have the most stable expression. According to Reid et al (2006), *EF1 *seemed to be the best reference gene (Figure 1) [6], according to Brunner et al (2004) *ACT *(Figure 2) [5], and with NormFinder *UBQ *[15]. Nevertheless, the differences in expression stability between the genes were very small and thus we conclude that all three genes are suitable to be used for normalizing gene expression in our study, even when used as reference gene alone. However, it has been shown that the use of several reference genes, which are not regulated under the different conditions between different treatments or tissue types, leads to an even higher reliability of quantitative gene expression studies [5].

### Importance of amplification efficiency for relative quantification

**Figure 3:**
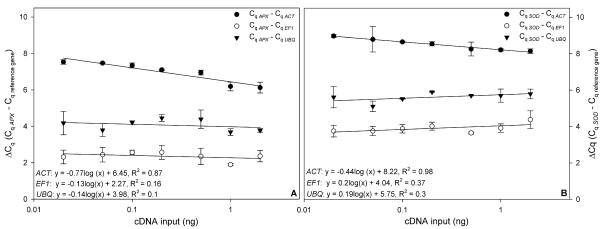
**Validation of the comparative Cq method**. Different amounts of cDNA were amplified using primer pairs for *ACT*, *EF1*and *UBQ *(reference genes), and *APX *and *SOD *(target genes). The ΔCq (Cq _target _- Cq _reference_) was plotted against the cDNA input, (A) *APX*, (B) *SOD*. n = 3.

A major point which has to be considered when using a relative quantification approach is the amplification efficiency. It has been shown that even minor variations in amplification efficiency can lead to considerable variation in the calculated gene expression [19]. Livak and Schmittgen (2001) presented an approach which depends on the assumption that after optimisation of Mg^2+ ^and primer concentrations the amplification efficiency of PCR is close to one [7]. Amplification efficiencies of target and reference genes have to be equal for the method to be valid. To test this, we prepared a dilution series of cDNA and plotted the ΔCq (Cq _target _- Cq _reference_) against the cDNA input (Figure 3). If the amplification efficiencies of two amplicons are similar, the slope of the regression of this plot is close to zero. This assumption is not true for all amplicons and therefore is a limitation of the method. For *APX *and *SOD *the assumption of same amplification efficiencies as the reference genes did not hold (Figure 3). However, Karlen et al (2007) [20] have found that the comparative Cq method is very robust and can therefore be used at least for approximate estimation of gene expression.

**Figure 4:**
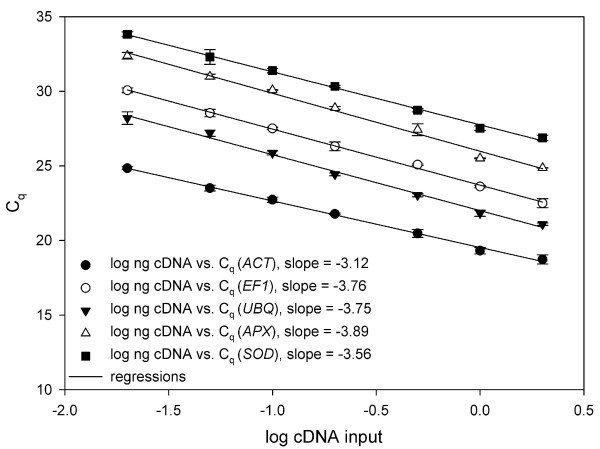
**Calculation of PCR efficiencies**. Real-time PCR efficiencies of reference (*ACT*, *EF1 *and *UBQ*) and target genes (*APX *and *SOD*) were determined. Cq was plotted against the log amount of cDNA input. Amplification efficiencies were calculated according to the equation E = 10^(-1/slope)^. *ACT*, actin; *APX*, ascorbate peroxidase; *EF1*, elongation factor 1; *SOD*, superoxide dismutase; *UBQ*, ubiquitin; n = 3.

For evaluation of amplification efficiencies two basic approaches have been described. One uses a dilution series of cDNA [10]. This approach is also used by the Pfaffl model [8]. The Cq values were plotted against cDNA input and efficiency calculated from the slope of the regression line according to the equation E = 10^(-1/slope) ^(Figure 4). In the investigated range, all amplicons showed relatively high amplification efficiencies, 2.09 for *ACT*, 1.84 for *EF1*, 1.85 for *UBQ*, 1.81 for *APX *and 1.91 for *SOD*. It is presumed that the efficiency is the same for all dilutions, however, when a cDNA sample is diluted, all compounds, which might inhibit the reaction, are also diluted and therefore can lead to higher amplification efficiencies in the diluted samples [19]. This problem can be circumvented by calculating the efficiency directly from the increase in fluorescence in each individual PCR sample, for which two mathematical models have been presented [11,12].

When using the approach from Liu and Saint (2002) [11], we found amplification efficiencies of 0.91 ± 0.01 for *ACT*, 0.81 ± 0.01 for *EF1*, 0.87 ± 0.02 for *UBQ*, 0.87 ± 0.02 for *APX *and 0.94 ± 0.02 for *SOD*. For this calculation, two arbitrary thresholds within the exponential phase have to be chosen. During evaluation of the method we found that minor variations in the choice of the thresholds can lead to large differences in the calculated amplification efficiency. When using the approach presented by Ruijter et al (2009) [12], we found amplification efficiencies of 1.97 ± 0.03 for *ACT*, 1.91 ± 0.01 for *EF1*, 1.94 ± 0.01 for *UBQ*, 1.95 ± 0.05 for *APX *and 1.92 ± 0.05 for *SOD*. In this method, the data were log-transformed before calculation of the efficiency from the log-linear phase, and in contrast to the Liu and Saint method [11], all data points within the log-linear phase were used, which makes it more reliable to detect the correct data points. Nordgård et al (2006) have shown that all approaches using the amplification plots of each individual reaction for calculation of amplification efficiencies produce large errors in quantification of gene expression [21]. They recommended using rather serial dilutions than individual reactions in order to increase precision. However, in recent years, the approach to use individual samples to determine amplification efficiencies has become more common in relative qPCR studies than using serial dilutions [22]. It has been shown that the most reliable approach is to assume the same amplification efficiency for all reactions with the same primer pair [23], to calculate the amplification efficiency from each individual amplification plot, check for outliers and use the mean efficiency for all samples [24].

### Evaluation of the quantification approaches and comparison of gene expression

**Figure 5:**
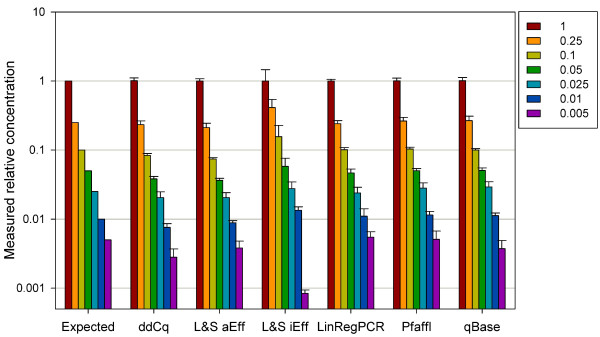
**Quantification of a cDNA dilution series**. Template amounts were calculated by applying the different quantification approaches and expressed relative to the undiluted sample. Average values ± standard errors for all tested amplicons are given. ddCq, comparative Cq method; L&S aEff, Liu & Saint (2002) method using average amplification efficiencies for each gene; L&S iEff, Liu & Saint (2002) method using individual amplification efficiencies for each reaction. n = 3.

We determined the template amount of a cDNA dilution series with all quantification approaches [20]. By comparing the results with the expected values we found that most of the tested approaches were able to detect the relative cDNA amount for all amplicons precisely (Figure 5). Only the approach using individual amplification efficiencies, i.e. the Liu and Saint method [11], showed significant deviations from the expected values. When applying the average amplification efficiency for each amplicon to the Liu and Saint method, the quality of the prediction of template amount was enhanced, confirming previous findings, that this is the most reliable approach for evaluation of amplification efficiency (Figure 5) [24].

**Figure 6:**
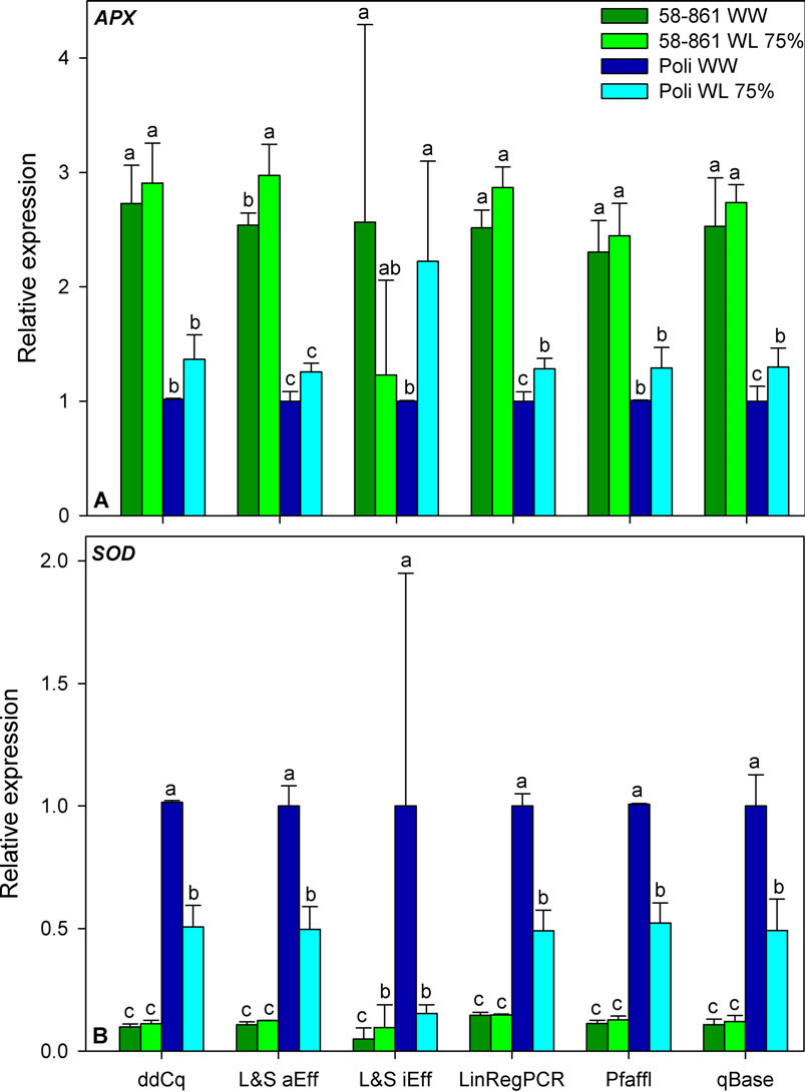
**Relative expression rates of the target genes *APX *and *SOD***. Relative gene expression rates of *APX *(A) and *SOD *(B) in roots of the poplar clones 58-861 and Poli were determined. Expression was calculated using all tested approaches for relative quantification, normalized to *ACT*, *EF1 *and *UBQ *transcript abundance and expressed in relation to WW Poli as calibrator sample. ddCq, comparative Cq method; L&S aEff, Liu & Saint method using average amplification efficiencies for each gene; L&S iEff, Liu & Saint method using individual amplification efficiencies for each reaction. *ACT*, actin; *APX*, ascorbate peroxidase; *EF1*, elongation factor 1; *SOD*, superoxide dismutase; *UBQ*, ubiquitin; WW, well-watered; WL 75%, water limited (75% less water than control), n = 3. Means ± standard errors indicated by the same letter are not significantly different (P ≥ 0.05) for each of the tested quantification approaches.

Accordingly, expression analysis of *APX *and *SOD *revealed consistent expression patterns in poplar roots under different watering regimes when using the comparative Cq method, LinRegPCR, the Liu and Saint method with average amplification efficiencies for each amplicon, the Pfaffl model and qBase. We found that relative expression of *APX *was higher in 58-861 than in Poli, but in both clones not influenced by the treatment. *SOD *expression was highest in well-watered Poli and reduced by the drought treatment, but was still higher than in 58-861 in both treatments (Figure 6). However, as primer mismatches have been shown to influence target amplification [25], the inter-genotype differences might possibly be due to the occurrence of SNPs within the primer binding sites. The results obtained with the approach using individual amplification efficiencies [11], showed high variability. Its poor performance in detecting the template amount of the cDNA dilution series (Figure 5) suggests that the gene expression patterns revealed by this method should be handled with caution. This approach revealed gene expression patterns contrasting to those obtained with the other applied approaches (Figure 6). This result shows that using a relative quantification approach which has not been validated for a certain study might even lead to biological misinterpretation of gene expression data. Interestingly, the results obtained with the comparative Cq method did not differ from the other methods, neither in predicting the template amount of the cDNA dilution series, nor in the gene expression patterns of *SOD *and *APX *in poplar roots. We would have expected a poorer performance of this method in our study, as the assumption of equal amplification efficiencies of target and reference genes was not fulfilled. However, previous studies comparing relative quantification approaches have shown that the comparative Cq method is very robust and can be used at least for approximate quantification in screening of large sample numbers [20].

## Conclusions

To summarize, we were able to show that the different approaches available for relative quantification of RT-qPCR data differ in their reliability, and that the results computed from the same dataset can differ considerably. The genes *ACT*, *EF1 *and *UBQ *selected to normalize expression of the target genes *APX *and *SOD *have been proven to be non-regulated in the different experimental conditions. In accordance with the literature, the approach calculating PCR efficiencies for each individual reaction [11] produced highly variable results. However, as it has been shown that the use of the mean amplification efficiency for each gene, computed from the individual reactions, is the most reliable approach, we conclude that the use of the Liu and Saint method is suitable to determine the amplification efficiencies, if the mean value for each primer pair is used for further analysis [24]. The good estimation of the template amount of a cDNA dilution series with known relative concentrations by the comparative Cq method, LinRegPCR, the Liu & Saint method with average amplification efficiencies for each amplicon, the Pfaffl model and qBase software suggest that these approaches produce reliable results. We do not offer a universal recommendation which approach should be used for relative gene expression studies, but we suggest that investigators should carefully evaluate different quantification approaches before using them in studies analysing gene expression.

## Methods

### Plant material, RNA extraction and cDNA synthesis

We used fine roots of two clones of black poplar (*Populus nigra *L.), Poli and 58-861, which had been subjected to different drought treatments [13,14]. Well-watered (WW) plants, which were watered to field capacity, were used as controls. Drought treatments were 50% water limitation (WL 50%) and 75% water limitation (WL 75%). All samples were used for evaluation of reference gene expression stability, whereas expression of the target genes was analysed only in WW and WL 75% samples. RNA was extracted using the Agilent Plant RNA Isolation Mini Kit according to the manufacturer's instructions (Agilent Technologies AG, Basel, Switzerland). RNA concentration was measured using a NanoDrop ND-1000 Spectrophotometer (NanoDrop Technologies, Wilmington DE, USA) in 1 μL volume. RNA quality was assessed using the Agilent Bioanalyzer (Agilent Technologies AG, Basel, Switzerland) with the Agilent RNA 6000 Pico Kit. First strand cDNA synthesis was performed with the QuantiTect Reverse Transcription Kit (Qiagen) using 200 ng total RNA according to the manufacturer's instructions. Reverse transcription was initiated using the RT-primer mix supplied with the kit, consisting of oligo-dT and random primers.

### Primer design and evaluation

Primers were designed using the Primer 3 software [26] for the amplification of gene fragments around 100 bp in length and an annealing temperature of 60°C. Sequences for primer design were downloaded from the *P. trichocarpa *v1.1 database at the Joint Genome Institute [27]. PCR was applied to test the specificity of the primers. An intron-spanning amplicon was chosen for *EF1 *in order to be able to verify absence of genomic DNA in the cDNA samples. PCR products were visualised after electrophoresis on 1% (w/v) agarose gels containing 0.02% (v/v) EtBr in 1× TAE buffer. Primer sequences and amplicon lengths are shown in Table 1[14].

**Table 1:** Primer sequences

Gene	Forward primer	Reverse primer	Amplicon size (bp)
Reference genes			
*ACT*	ACC CTC CAA TCC AGA CAC TG	TTG CTG ACC GTA TGA GCA AG	105
*EF1*	AAG CCA TGG GAT GAT GAG AC	ACT GGA GCC AAT TTT GAT GC	101
*UBQ*	CGT GGA GGA ATG CAG ATT TT	GAT CTT GGC CTT CAC GTT GT	99
Target genes			
*APX*	TCT TGC GAG GAA GTG AAG GT	AAT GGT TGG ACC TCC AGT GA	100
*SOD*	GGG TCT CGT CCA ACA CAC TT	AGC CAT GGC GAT AGA TTG AC	96

Primer sequences and amplicon sizes for the reference genes *ACT*, *EF1 *and *UBQ *and the target genes *APX *and *SOD *[14].

### RT-qPCR

RT-qPCR reactions of 15 μL total volume contained 7.5 μL 2× FastStart Universal SYBR Green Master Mix (Roche, Basel, Switzerland), 5 μM of forward and reverse primers and 1 μL of 1:10 or 1:100 diluted cDNA. RT-qPCR was performed on an ABI 7500 Fast real-time PCR system (Applied Biosystems) with the following conditions: 10 min 95°C initial denaturation; 40 × 15 sec 95°C denaturation, 60 sec 60°C primer annealing/elongation. The fluorescence was recorded during the annealing/elongation step in each cycle. A melting curve analysis was performed at the end of each PCR by gradually increasing the temperature from 60 to 95°C while recording the fluorescence. A single peak at the melting temperature of the PCR-product confirmed primer specificity. To be able to compare between different runs, we used a fixed fluorescence threshold for derivation of the Cq value for all runs. We performed three technical replicates for each of three biological replicates per clone/treatment combination to evaluate the relative quantification approaches.

### Data analysis

Expression stability of the reference genes was tested by calculating the standard deviations of the Cq for each gene between all treatments [6]. To confirm expression stability, it was also tested by a second approach which uses a regression of Cq against all sample types [5], and additionally, the NormFinder application for Microsoft Excel was used [15]. Dilution series for all primer/sample combinations were prepared to evaluate amplification efficiencies. For relative quantification of our gene expression data, we tested five widely used approaches.

The comparative Cq method is based on the differences in Cq between target and reference genes and normalizes gene expression to a calibrator sample [7]. We tested for differences in amplification efficiencies of target and reference genes by producing a cDNA dilution series and plotting the ΔCq (Cq _reference gene _- Cq_target gene_) against the dilution, which should result in a slope of the regression line of close to zero. In a second step, the difference between the samples and a calibrator ΔΔCq (ΔCq _sample _- ΔCq _calibrator_) was calculated and used to determine the relative expression rate (r). In an ideal reaction, the amplification efficiency is close to one, which leads to the equation r = 2^-ΔΔCq ^for relative quantification of gene expression. qBase software depends on the same principle as the comparative Cq method, but allows to include correction for amplification efficiencies and multiple reference genes for normalization [9]. For quantification with qBase we used the mean amplification efficiencies calculated by LinRegPCR [12].

Pfaffl presented a mathematical model which also takes into account that the amplification of different genes may have different efficiencies [8]. Amplification efficiency (E) was calculated from the plot of the Cq values against cDNA input according to the equation E = 10^(-1/slope) ^[10]. The relative expression ratio of a target gene in comparison with a reference gene was calculated according to the equation r = E_target_^ΔCq target (calibrator - sample)^/E_reference_^ΔCq reference (calibrator - sample)^.

In the method presented by Liu and Saint the amplification efficiency is determined from the increase in fluorescence (R) of each individual reaction during PCR [11]. We selected two arbitrary thresholds (R_A _and R_B_) within the exponential phase of the PCR curve from a graph of the amplification plots (log fluorescence against cycle number). Efficiency was determined according to the equation E = (R_B_/R_A_)^1/(CqB-CqA) ^- 1. For quantification of gene expression we used two approaches: first, we used individual amplification efficiencies for each sample, and second, we used the average efficiency for each amplicon. The starting content of the target sequence (R_0_) was calculated using the equation R_0 _= R_Cq_/(E+1)^Cq ^and normalized to the starting amount of the reference gene.

With LinRegPCR software we determined gene expression from the slopes of the amplification curves [12]. The data were log-transformed and a regression line determined from the log-linear phase of PCR. The starting concentration of the template was then directly computed from the intercept of the regression line.

With all quantification approaches we quantified a cDNA dilution series with known relative concentrations and compared the results with the expected values [20].

One-way ANOVA was applied using SPSS 16.0 for Windows statistical software package (SPSS Schweiz AG, Zurich, Switzerland) to determine differences in gene expression between clones and treatments. All results are presented as mean values ± standard errors.

## Authors' contributions

NR carried out the molecular studies and data analysis, and drafted the manuscript. BF conceived the experimental design and helped to draft the manuscript. Both authors read and approved the final manuscript.
